# De novo leaf and root transcriptome analysis identified novel genes involved in Steroidal sapogenin biosynthesis in *Asparagus racemosus*

**DOI:** 10.1186/1471-2164-15-746

**Published:** 2014-08-30

**Authors:** Swati Upadhyay, Ujjal J Phukan, Sonal Mishra, Rakesh Kumar Shukla

**Affiliations:** Biotechnology Division, Central Institute of Medicinal and Aromatic Plants, P.O. CIMAP, Near Kukrail Picnic Spot, Lucknow, U.P India

**Keywords:** *Asparagus racemosus*, saponin, Transcriptome, *De novo* assembly, Unigenes

## Abstract

**Background:**

Saponins are mainly amphipathic glycosides that posses many biological activities and confer potential health benefits to humans. Inspite of its medicinal attributes most of the triterpenes and enzymes involved in the saponin biosynthesis remains uncharacterized at the molecular level. Since the major steroidal components are present in the roots of *A. racemosus* our study is focussed on the comparative *denovo* transcriptome analysis of root versus leaf tissue and identifying some root specific transcripts involved in saponin biosynthesis using high-throughput next generation transcriptome sequencing.

**Results:**

After sequencing, *de novo* assembly and quantitative assessment, 126861 unigenes were finally generated with an average length of 1200 bp. Then functional annotation and GO enrichment analysis was performed by aligning all-unigenes with public protein databases including NR, SwissProt, and KEGG. Differentially expressed genes in root were initially identified using the RPKM method using digital subtraction between root and leaf. Twenty seven putative secondary metabolite related transcripts were experimentally validated for their expression in root or leaf tissue using q-RT PCR analysis. Most of the above selected transcripts showed preferential expression in root as compared to leaf supporting the digitally subtracted result obtained. The methyl jasmonate application induces the secondary metabolite related gene transcripts leading to their increased accumulation in plants. Therefore, the identified transcripts related to saponin biosynthesis were further analyzed for their induced expression after 3, 5 and 12 hours of exogenous application of Methyl Jasmonate in tissue specific manner.

**Conclusions:**

In this study, we have identified a large set of cDNA unigenes from *A. racemosus* leaf and root tissue. This is the first transcriptome sequencing of this non-model species using Illumina, a next generation sequencing technology. The present study has also identified number of root specific transcripts showing homology with saponin biosynthetic pathway. An integrated pathway of identified saponin biosynthesis transcripts their tissue specific expression and induced accumulation after methyl jasmonate treatment was discussed.

**Electronic supplementary material:**

The online version of this article (doi:10.1186/1471-2164-15-746) contains supplementary material, which is available to authorized users.

## Background

*Asparagus racemosus* (*A. racemosus*) is an important medicinal plant which has been used worldwide. The *Asparagus* genus (Asparagaceae) has over 300 species which are widely distributed in temperate and tropical regions including India, China, Korea and Japan [1]. Its medicinal properties are reported in traditional systems of medicine such as Ayurveda, Siddha and Unani [2]. The plant is a spinous under shrub with tuberous short rootstock bearing numerous succulent tuberous roots (30–100 cm long and 1–2 cm thick) that are silvery white or ash-colored externally and white internally [3].

The tuberous root of *A. racemosus* is used in traditional Indian medicine for the treatment of diverse ailments, including dysentery, tumors, inflammations, neuropathy, nervous disorders, bronchitis, hyperacidity, certain infectious diseases [4], conjunctivitis [5], chronic fevers, and rheumatism [6]. Pharmacological studies with animals have manifested the potency of *A. racemosus* extract as an antioxidant [7], antianaphylactic [8], adaptogen [9], antistress [10], antiulcer [11], antidiarrhoeal [12], antibacterial [13], antitussive [14], molluscicide [15], radioprotective agent [16], and as a substrate for inulinase production [17] with the biggest focus being on its ability in modulating the immune system [18]. One human trial confirmed the herb’s potency in treating dyspepsia [19]. Due to its vast medicinal properties it is well known as an antispasmodic, aphrodisiac, demulcent, diuretic, galactagogue and refrigerant in Indian medicine (Ayurveda) [20].

A limited number of steroidal saponins have been reported previously from the roots of *A. racemosus*, with the major ones being shatavarins I, IV, V [21] and immunosides [22]. Taxol, a steroidal saponin of *Taxus bravifolia* bark is currently being used in cancer chemotherapy [23]. Diosgenin, (25R)-Spirost-5-en-3β-ol, is a steroidal sapogenin isolated from the plants [24]. It is very useful in pharmaceutical industries as a natural source of steroidal hormones. Recent studies have found that steroidal sapogenin can be absorbed through the gut and plays an important role in the control of cholesterol metabolism [25]. Other authors have reported that it has estrogenic effects [26] and antitumor activity [27]. McAnuff et al. (2002) reported that steroidal sapogenins were effective agents for the treatment of hypocholesterolemia, a disorder often associated with diabetes [28].

The diverse structures of steroidal saponins make them valuable in commercial applications, as well as in drugs and medicines. It is thought that steroidal sapogenins are biosynthesized from cholesterol via a series of oxygenation and hydroxylation steps, and that they are then glycosylated to form steroidal saponins [29]. The mevalonate pathway or HMG-CoA reductase pathway or mevalonate-dependent route or isoprenoid pathway, is an important cellular metabolic pathway present in all higher eukaryotes and many bacteria. The mevalonate pathway is responsible for the biosynthesis of numerous essential molecules including prenyl groups, coenzyme Q, dolichol, and sterols such as cholesterol [30]. The knowledge of steroidal biosynthesis is derived from studies of cholesterol production through Acetate → Mevalonate → Isopentenyl pyrophosphate → Squalene pathway. The biosynthesis of cholesterol involves cyclization of aliphatic triterpene-squalene [31]. Cholesterol has been found to be an effective precursor for diosgenin [32]. However, the enzymes and genes involved in the biosynthesis of these complex molecules are largely uncharacterized. Only a limited number of enzymes have been identified and characterized that play an important role in the modification of the saponin backbone structure. This includes enzymes like cytochrome p450 monooxygenase, uridine diphosphate glycosyltransfearse (TGTs) and other enzyme [33]. Secondary metabolic pathway genes are more diverse in comparison with those involved in primary metabolism. The advent of high-throughput sequencing technologies has permitted new approaches to explore functional genomics, including the direct sequencing of complementary cDNA generated from messenger and structural RNAs (RNA-seq). Transcriptome analysis followed by identification of potential candidate genes involved in the secondary metabolic pathway will lead to a better understanding of biosynthesis, its regulation and chemical diversity of secondary metabolites.

*A. racemosus* is well known medicinal Ayurvedic plant listed in the British Pharmacopoeias. Here we have performed the pair end transcriptome sequencing of root and leaf tissue of *A. racemosus*. Paired end sequencing improves the efficiency of *de novo* assembly and also increases the depth of sequencing. Using different assembly programs we have reported the most appropriate, assembled set of non redundant transcripts identified. The resulting assembled transcripts were functionally annotated, and the transcripts involved in secondary metabolic biosynthetic pathway were analyzed. The occurrence and fold induction of differentially expressed transcripts in leaf or root was further analyzed using digital gene expression analysis followed by their validation using q-RT PCR analysis. Sixteen putative transcripts involved in secondary metabolite biosynthesis were analyzed for their relative expression in response to Methyl jasmonate treatment. This data will lead to the advancement in the understanding of saponin biosynthetic pathway. The enzyme/transcript identified will serve the purpose of engineering of steroidal saponin biosynthesis in other medicinal plants.

## Results

### Sequence quality control and *de novo*assembly

**Figure 1:**
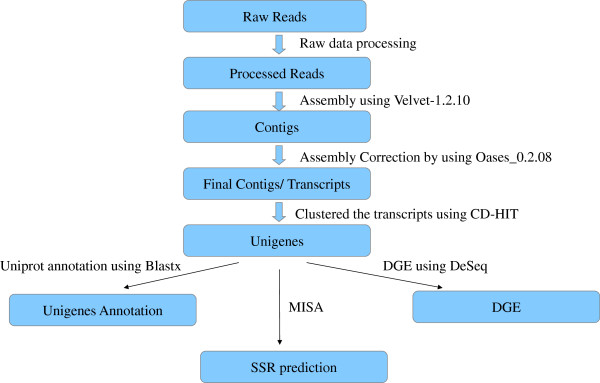
**Overall strategy of leaf versus root Illumina transcriptome sequencing, data analysis and annotation of** ***Asparagus racemosus*** **unigene obtained.**

cDNA libraries prepared from RNA isolated from leaf and root tissues of *A. racemosus* were sequenced and a total of 54893366 and 59911356 raw reads were generated comprising 5501317136 and 6007075848 nucleotide bases from Illumina GAII Analyzer/454 GS FLX/5500 SOLiD System. Sequencing accounts for approximately 6.79 Gb and 7.43 Gb of data for leaf and root samples of *A. racemosus* (CIM-Shakti) respectively. Raw reads were subjected to quality control by using SeqQC-V2.1. Low quality reads which contain adapters or unknown or low quality bases were discarded. Finally, in total 54893366 and 59911356 reads were generated from leaf and root samples respectively. The quality of a *de novo* assembly is dependent on many factors, such as the selection of an assembler followed by parameters like Hash length, N50 Value and coverage. The quality of the transcriptome assembly may also be determined by the assembly software that is employed to assemble the short read data. Velvet assembler was used to produce a preliminary fragmented assembly, containing the mapping of the reads onto a set of contigs. After reading the contigs produced by Velvet, Oases proceeds to correct them again with a set of dynamic and static filters. A total of 28390071(51.7185829%) reads from leaf (Hash length = 47; N50 Value = 462) and 36299342 (60.58841666%) reads from root (Hash length = 43; N50 Value = 762) sample were selected for assembly by velvet programme and 45877643 (83.57593338%) reads from leaf (Hash length = 47; N50 Value = 1522) and 55904673(93.31231461%) reads from root sample (Hash length = 43; N50 Value = 2179) were selected for assembly by using Oases program. The overall strategy is schematically represented in Figure 1. A total of 173681 contigs for leaf and 83561 contigs for root are generated by using Velvet programme and 107080 transcripts for leaf and 78684contigs for root are obtained by using Oases programme. In both samples, the average length of contig range from 200 bases to more than 1200 bases (Additional file 1: Figure S1). Average contig length by using Oases programme was more (1,043.2 ± 813.6 for leaf and 1,463.4 ± 1,313.2 for root) than the average contig length generated by using Velvet programme (426.5 ± 311.0 for leaf and 602.1 ± 525.9 for root). The sequences from both the libraries were deposited in the NCBIs Short Read Archive under the accession number SRA1156272.

### Functional annotation and classification

**Figure 2:**
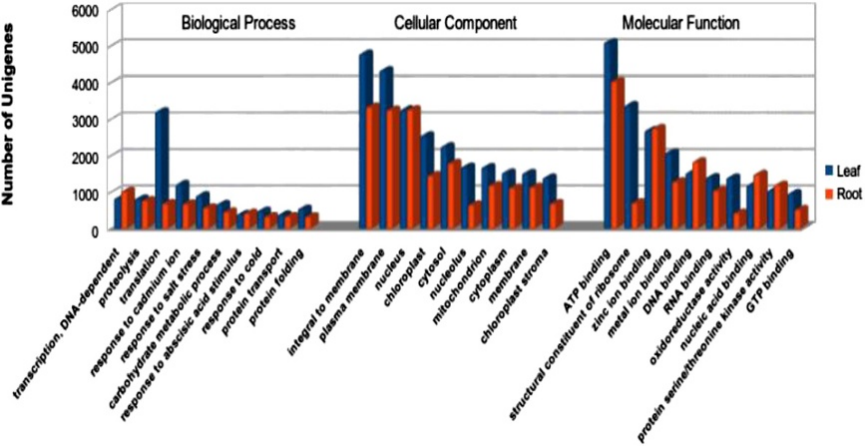
**Histogram presentation of Gene Ontology classification.** The results are summarized in three main categories, Biological process, Cellular component, and Molecular function. The y-axis indicates the percentage of genes in a category, and the x-axis shows the number of genes. Leaf and root tissue are marked with blue and red colour.

For the validation and annotation of the assembled unigenes, all the assembled unigenes were searched against the NCBI non-redundant (nr) and Swissprot protein databases using BLASTX program with an E-value threshold of 1^E-5^. Among 126861 unigenes 1325 (1.04%) were found similar with *Arabidopsis* and 3402 (2.68%) unigenes were found similar with *Liliopsida*. A total of 76725 (60.47%) unigenes were found in both databases, while 45409 (35.79%) unigenes were unidentified (Additional file 1: Figure S2). Based on Nr annotations, we used the Gene Ontology (GO) classification system to classify the possible functions of the unigenes. Almost all the unigenes were successfully assigned to at least one GO term annotation. The unigenes were then classified into three main categories, biological processes, cellular components, and molecular function (Figure 2).

**Figure 3:**
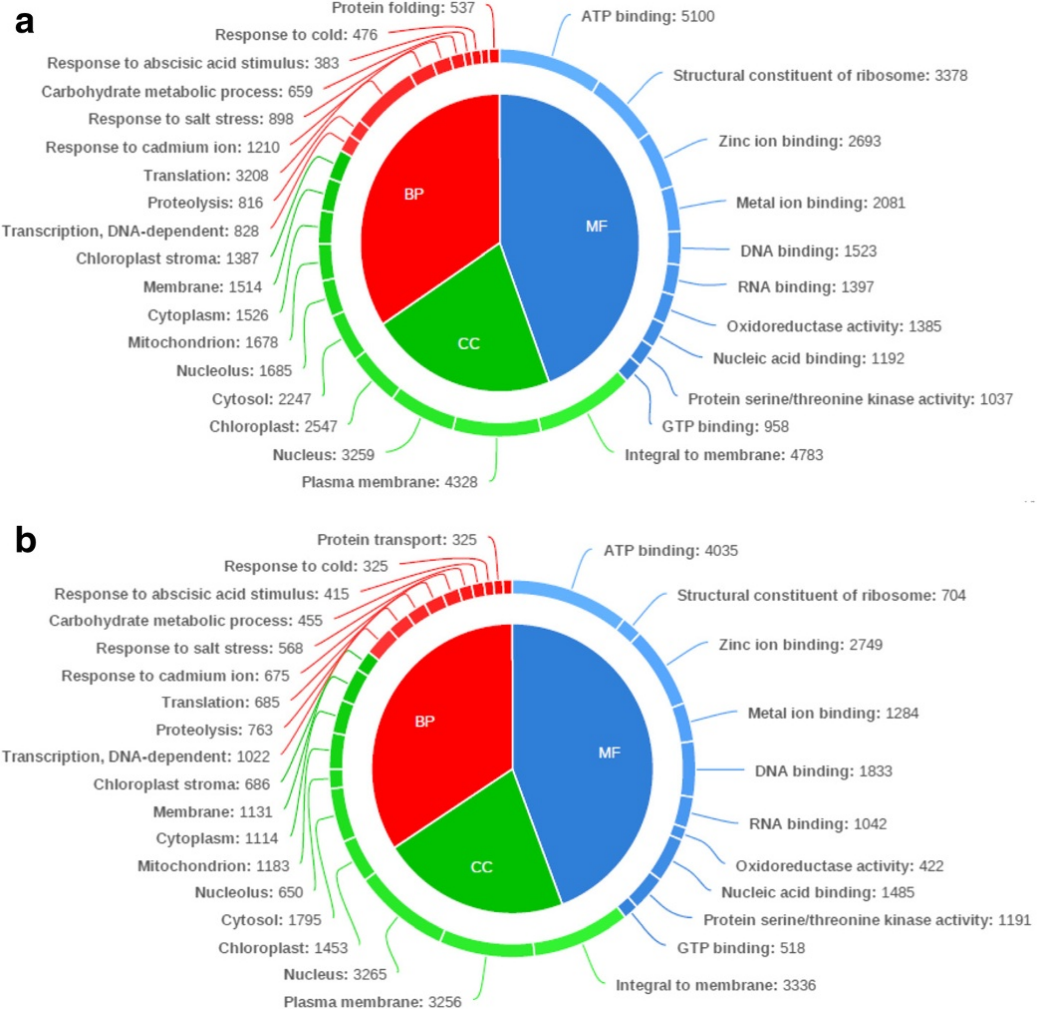
**Top ten most represented GO terms in each of the three GO domains. (a)** In leaf sample **(b)** In root sample

The category of biological processes (leaf and root) consisted of total 3990 GO terms, which were assigned to 89421 unigenes. The cellular components (leaf and root) category consisted of total 878 GO terms, which were assigned to 81348 unigenes. The category of molecular functions (leaf and root) consisted of total 3229 GO terms, which were assigned to 99868 unigenes. In leaf, for biological process, the top three largest categories were, “translation” (3208), “response to cadmium ion” (1210), and “response to salt stress” (898). For cellular components, the top three largest categories were, “integral to membrane” (4783), “plasma membrane” (4328), and “nucleus” (3259). For molecular function, the top three largest categories were, “ATP binding” (5100), “structural constituents of ribosomes” (3378), and “zinc ion binding” (2693). In root, for biological process, the top three largest categories were, “transcription, DNA dependent” (1022), “proteolysis” (763), and “translation” (685). For cellular components, the top three largest categories were, “integral to membrane” (3336), “nucleus” (3265), and “plasma membrane” (3256). For molecular function, the top three largest categories were, “ATP binding” (4035), “zinc ion binding” (2749), and “DNA binding” (1833) (Figure 3a, b).

### Identification of Simple Sequence Repeats (SSRs) and their nature of repeat present in secondary metabolic pathway related genes

SSR markers are the most important molecular markers in plants and have proven to be a valuable tool for various applications in genetics and plant breeding. Therefore, to develop a novel set of functional SSRs all of the 185764 unigenes generated in this study were used to determine potential microsatellite motifs using MIcroSAtellite (MISA) software (http://pgrc.ipk-gatersleben.de/misa). Total number of SSRs found in root tissues was more than SSRs found in leaf tissues. 18107 SSRs in 15338 sequences were recognized in leaf samples (Additional file 2: Table S3). Root samples showed 26733 SSRs in 21369 sequences (Additional file 3: Table S4). Number of sequences containing more than 1 SSR in leaf was 3385 and in root was 5976. Tri-nucleotide repeats were the most abundant SSR motif in leaf tissues followed by di-nucleotide, tetra-nucleotide, penta-nucleotide and hexa-nucleotide motifs (Additional file 1: Figure S3a) but in root tissues di-nucleotide repeats were the most abundant SSR motif followed by tri-nucleotide, tetra-nucleotide, penta- nucleotide and hexa-nucleotide motifs (Additional file 1: Figure S3b). The number of compound SSRs presents in leaf and root samples were 1450 and 2462 respectively. We have checked for the presence of these SSRs motifs in transcripts involved in steroidal sapogenin biosynthetic pathway. HMG Co-A reductase shows both compound SSRs, mono and tri-nucleotide repeats at sequence level. In addition to this Methylsterol Monooxygenase and Cycloartenol C-24 Methyltransferases have mononucleotide repeats in its sequences on the other hand HMG-COA-synthase was found to have di-nucleotide repeat (Additional file 3: Table S5).

### Metabolic pathway analysis by KEGG

The Kyoto Encyclopedia of Genes and Genomes (KEGG) Pathway database records the network of molecular interactions in the cells and variants of them specific to particular organisms. Pathway-based analysis helps us to further understand the biological functions and interactions of genes. To further analyze the transcriptome of *A. racemosus*, all the unigenes were analyzed in KEGG pathway database. First, based on a comparison against the KEGG database using BLASTX with an E-value threshold of 10^−5^, 162 unigenes in leaf and 156 unigenes in roots were found to have significant matches in the database and were assigned to 124 KEGG pathways that are related to secondary metabolite biosynthesis (Additional file 4: Table S2). It was also observed that there were 50 unigenes in leaf sample and 61 unigenes in root sample, encoding enzymes that were involved in triterpenoid biosynthesis (Additional file 3: Table S8).

### Identification and upregulation of Terpenoid backbone biosynthesis genes

Precursor molecules for steroidal saponin biosynthesis belong to the terpenoid backbone, which utilizes isoprenoids synthesized via Mevalonate as well as MEP pathway. In our transcriptome data there were 28 unigenes in leaf and 29 unigenes in root, related to terpenoid backbone biosynthesis. Almost all of the genes encoding the enzymes involved in terpenoid backbone biosynthesis were present in our data. We have identified 20 enzymes (unigenes) in root and 16 enzymes (unigenes) in leaf related to MVA pathway and 9 enzymes in root and 7 enzymes in leaf related to Mevalonate pathway of terpenoid biosynthesis. In most cases, more than one unigenes were assigned to the same enzyme. Such unigenes may represent different fragments of a single transcript, different members of a gene family, or both (Additional file 3: Table S8).

### Identification and expression analysis of transcripts related to steroidal sapogenin and their specific expression in root of *Asparagus racemosus*

To identify genes with different expression levels in root as compared to leaf (as control), initially we used the RPKM method (Reads Per kb per Million reads) to calculate the expression levels of the unigenes. The log_10_ RPKM values were determined which range from less than 0.5 to more than 4.0 with an average of 2.1304 for root and 1.9286 for leaf (Additional file 1: Figure S4). Fold change values were also given in addition to their RPKM values which indicated that most of the genes are expressed with a fold change value ranging between 2–6 (Additional file 1: Figure S5). We used the ratio of RPKMs to calculate the fold-change in the expression (DGE) of each gene in two samples simultaneously. We observed 2934 differentially expressed transcripts by digital gene expression analysis in which 781 (26.61%) transcripts were up regulated in root with log 2 Fold Change value of more than 4 (Additional file 3: Table S6). After BLAST-X search of these Root specific transcripts found to be up regulated in DGE data, we obtained number of them to be functionally involved in Steroidal saponin biosynthetic pathway, such as Cytochrome P450s which showed a fold change value ranging between 277.48 to 2203.81, Methylsterol monooxygenases with a fold change value ranging between 154.54 to 949.06 and UDP-glycosyltransferase with a fold change of 729.15 (Additional file 3: Table S6). A total of 1640 unigenes were identified to be involved in transcription, including DGEs (407 up-regulated and 351 down-regulated) (Additional file 1: Figure S6a). These transcription factors were distributed to 69 families. Some of the largest TF families identified in leaf and root tissues of *A. racemosus* were the CCAAT-box binding transcription factors i.e. CCAAT [34], followed by the AP2-EREBP family [35], MYB family [36], WRKY family [37], C3H, HB [38], SNF2 [39], Orphans [40] and NAC [41] transcription factor super family. Digital gene expression of transcription factors in leaf and root tissues showed that 40 transcripts of MYB superfamily followed by 33 transcripts of AP2-EREBP and 23 transcripts of bHLH superfamily were highly upregulated in root (Additional file 1: Figure S6b).

**Figure 4:**
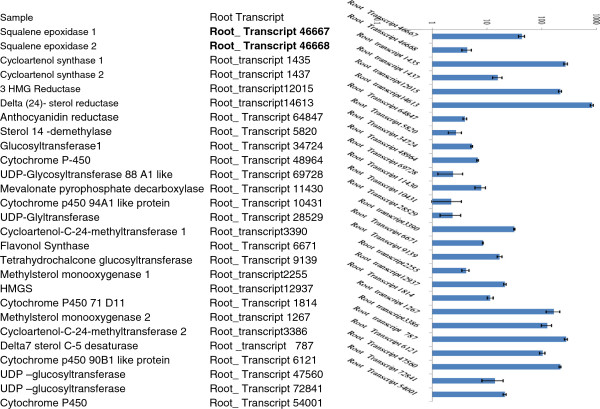
**Validation of selected twenty seven up regulated transcripts in root as compared to leaf (control) by using qRT-PCR analysis.** These all transcripts were related to secondary metabolite biosynthesis. Actin was used as the internal reference gene and the relative abundance of each gene transcript in roots and leaf tissue was compared. The data represents three independent biological and experimental replicates performed with standard deviations.

To confirm that the unigenes from sequencing and computational analysis were indeed expressed and also to analyze the differential gene expression profile between leaf and root tissues, twenty seven unigenes from the above 781 root specific transcripts related to secondary metabolite biosynthesis were chosen for qRT-PCR analysis. All of these genes showed up regulation under control condition in root as compared to leaf control (Figure 4). Among the highly expressed transcripts in our validation experiment are Cytochrome p 450 90B1 like protein (Root_ Transcript 6121; around 100 fold), UDP –glucosyltransferase (Root_ Transcript 47560; More than 200 fold), Methylsterol monooxygenase (Root_transcript 1267; More than 200 fold), Delta7 sterol C-5 desaturase (Root _transcript 787; More than 250 fold ) , Cycloartenol-C-24-methyltransferase (Root_transcript 3386; around 150 fold), 3 HMG Reductase (Root_transcript 12015; More than 200 fold ), Delta (24)- sterol reductase (Root_transcript 14613; more than 800 fold ) and Cycloartenol synthase (Root_transcript 1435; around 300 fold). The real time expression data further validated the presence of these twenty seven selected transcripts based on DGE data for their specific expression in root tissue in comparison with leaf. Out of above 27 transcript Methylsterol Monooxygenase, Delta7 sterol C-5 desaturase, Cycloartenol-C-24-methyltransferase, 3 HMG Reductase, Delta (24)- sterol reductase, and Cycloartenol synthase are involved in Steroidal sapogenin biosynthetic pathway. Modifications of saponins are shown to be important for their biological activity. We have also checked the level of identified UDP glycosyltransferase which was found to be specifically present in root rather than leaf tissue (Figure 4).

Steroidal sapogenins are synthesized from cholesterol in several plants but not much information is available about biosynthetic pathways of steroid saponins and related metabolites apart from the knowledge that cholesterol and sitosterol are their cycloartenol-derived precursors. Three pathways namely glycolytic pathway, Mevalonate pathway and steroid biosynthesis pathway involved in steroidal saponin biosynthesis. Integrating these three pathways we arrived at a conclusion that steroidal sapogenins may be formed from squalene 2, 3-oxide in two ways, either from lanosterol via the formation of cholesterol, or from cycloartenol via the formation of sitosterol. The enzymes involved in steroidal sapogenin biosynthesis that were found to be up regulated in root are listed in Additional file 3: Table S7 with their transcript ID and EC numbers. In most cases, more than one unigenes were assigned to the same enzyme. Such unigenes may represent different fragments of a single transcript, different members of a gene family, or both.

### Quantitative Real-Time PCR (q RT-PCR) analysis of saponin related gene transcripts in response to methyl jasmonate treatment

**Figure 5:**
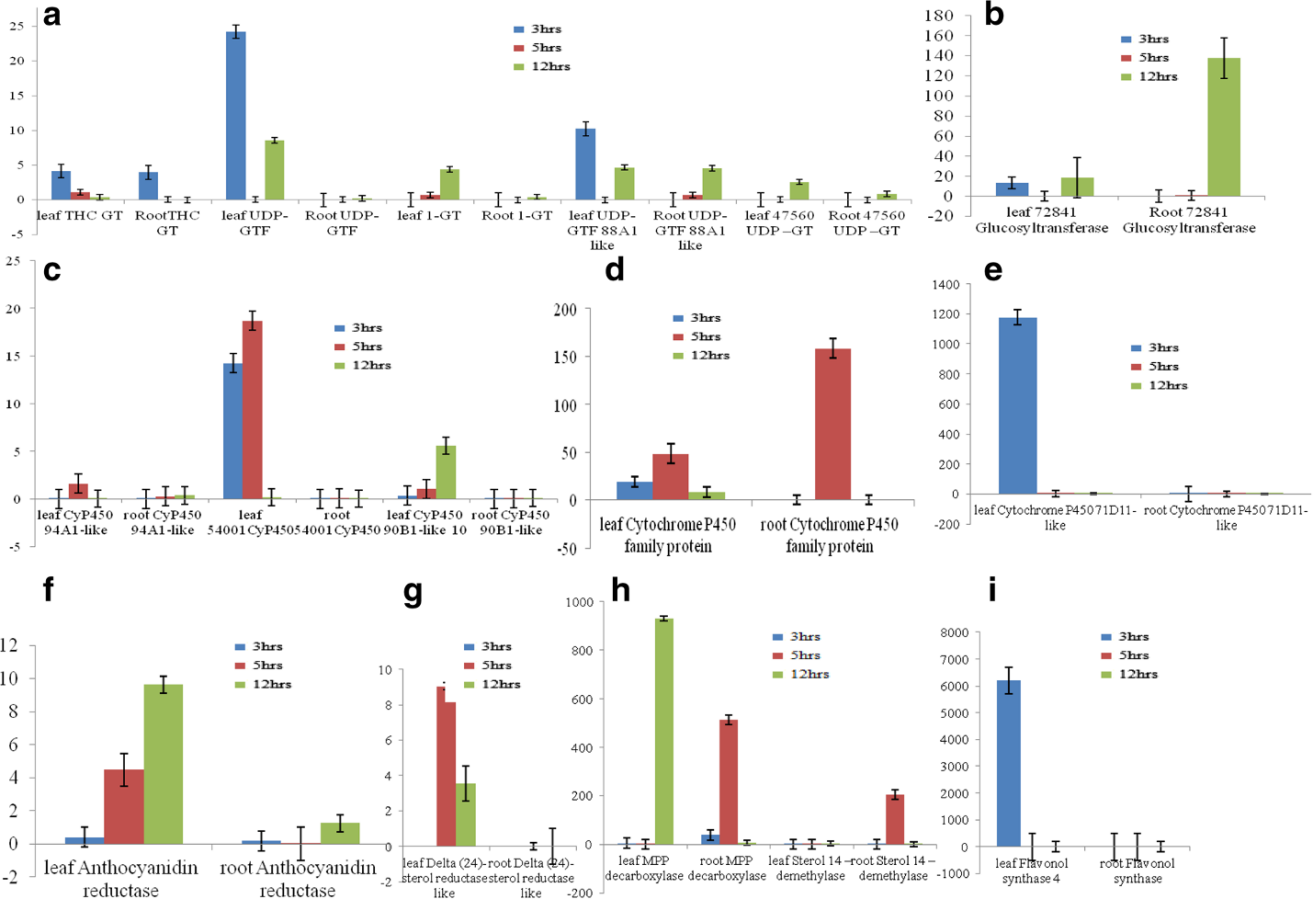
**Relative expression levels of secondary metabolism related gene transcripts in** ***A. racemosus*** **root and leaf tissues after 3, 5 and 12 hours of MeJA treatment. (a)** and **(b)** Represents the q-RT-PCR analysis of glucosyltransferase (GT) and glycosyltransferase (GTF) enzymes identified in transcriptome analysis **(c)**, **(d)** and **(e)** represents the expression of cytochrome-p-450 enzymes identified after transcriptome assembly **(f)**, **(g)**, **(h)**, **(i)** represents the q-RT PCR analysis of other saponin related gene transcripts identified. Total RNA was extracted from root and leaf of one month old plant and expression level of identified transcript was analyzed by q-RT PCR. Actin was used as the internal reference gene and the relative abundance of each gene transcript in root and leaf tissue was compared. The data represents three independent biological and experimental replicates performed with standard deviations.

Methyl Jasmonate acts as an important elicitor, signaling molecule and also regulates the activity of various enzymes related to the secondary metabolic pathway. Methyl jasmonate induces the saponin biosynthesis transcripts leading to increased accumulation of saponin. We have selected 16 transcripts from root, related to secondary metabolite biosynthesis specifically to saponin biosynthesis and checked their relative expression after 3, 5 and 12 hours of exogenous application of MeJA treatment in leaf and root tissues respectively (Additional file 5: Table S1). All the transcripts showed differential accumulation in response to MeJA at different time points. Transcripts of THC-glucosyltransferase (GT), UDP- glycosyltransferase (GTF) 88 A1 like and 72841 glycosyltransferase (GTF) showed induced accumulation in root as well as in leaf while UDP glycosyltransferase (GTF), 1- glucosyltransferase (GT) and 47560- glucosyltransferase (GT) showed induced accumulation only in leaf tissue after MeJA treatment (Figure 5a and b). Except CyP450 family transcript, rest of the transcript of CyP450s for example CyP450 94 A1-like, 54001 CyP450, CyP450 90-B1 like and CyP450 71 D11-like transcripts showed increased accumulation in leaf only (Figure 5 c, d and e). Anthocyanidin reductase showed induced accumulation in leaf tissue while in root there was no significant increase (Figure 5f). Delta (24) sterol reductase showed enhanced accumulation in leaf (Figure 5 g) while transcript of sterol 14-demethylase showed induced expression in leaf as well as in root but with a higher fold induction (Figure 5 h). The expression level of Mevalonate Pyrophosphate Decarboxylase in leaf is more than root (Figure 5 h) and the expression level of flavonol synthase is more in leaf than root (Figure 5i).

## Discussion

### *De novo*assembly and functional annotation

*A. racemosus* is an important plant used for medicinal and ornamental purposes. Despite pharmacological importance, the transcriptomic and genomic data of *A. racemosus* are very limited that are available in the National Center of Biotechnology Information (NCBI) database. In NCBI only 97 ESTs and 22 nucleotide sequences are available from *A. Racemosus* [42]. Characterization of transcriptome is especially important for such a plant species which have a very large genome and present a great challenge for whole genome sequencing. Due to the immense pharmacological importance of saponins found in roots of *A. racemosus, de novo* transcriptome analysis of leaf vs root tissue was done.

Because of the development of an array of novel assembly methods, short read assembly has become cost-effective. With recent improvements in assembly programs that can effectively assemble relatively short reads, coupled with the great advantage of paired-end sequencing, the short read sequence data generated for transcriptomes or whole genomes have been assembled *de novo* very successfully for species such as maize [43], soybean [44], giant panda [45], carrot [46], rubber tree [47], peanut [48] safflower [49], sweet potato [50], etc. Therefore, *de novo* sequencing and assembly of transcriptome or genome have been successfully used for model [51] and non-model [52] organisms. In this study, more than 57.41 million high-quality reads were used to assemble the leaf and root transcriptome of *A. racemosus*. The assembly result indicated that the mean length of unigenes was 1200 bp, which was longer than the results shown in previous studies. We obtained more than 100% HQ bases for both root and leaf samples which reflect the high quality sequencing run. Low quality bases as well as the presence of adapters in reads could interfere with the assembly process resulting in misassemblies or truncated contigs. Hence, low quality bases and adapter sequences were trimmed before proceeding with further analysis. These results suggested that the transcriptome sequencing data from *A. racemosus* were effectively assembled, which was further validated by the high proportion of unigenes matched with known proteins. The observed N50 value was higher which further suggests a better assembly.

The best hit for each unigene queried against the KEGG and NCBI Nr database was utilized to assign functional GO annotation in terms of biological process, molecular function and cellular component groups. The large number of diverse GO assignments to unigenes highlights the diversity of genes likely represented in *Asparagus* leaf and root transcriptome data. Mapping these unigenes on to KEGG, we had identified large number of unigenes involved in biosynthesis of various secondary metabolites. The unigenes without hits probably belonged to untranslated regions, noncoding RNA, short sequences not containing a protein domain or assembly mistakes. The functions of the identified genes cover various molecular function categories, and the well-represented categories included plasma membrane, integral membrane, nucleotide binding, structural component of ribosomes and nuclear proteins. The sequences encoded a broad set of transcripts represented within the cellular component category which indicates the need of a large number of transcripts for cellular structure and maintenance. On the basis of the annotation, we found the genes encoding all of the enzymes involved in biosynthesis of triterpenoid backbone, mostly in roots (including MVA and MEP pathways).

### Potential candidate genes involved in steroidal saponin biosynthesis

Steroidal sapogenins are synthesized from cholesterol in several plants [53], through isoprenoid biosynthetic pathway. Cytosolic isoprenoids are synthesized from acetyl CoA through intermediate formations of mevalonate, isopentanyldiphosphate, dimethylallyldiphosphate, isopentenyl diphosphate, geranyldiphosphate, faresnyl diphospahte, squalene, cycloartenol and leads to steroidal sapogenin in broad view.

Most of the known enzymes involved in the MVA pathway for triterpene steroidal biosynthesis were found to be specifically expressing in root in comparision with leaf transcriptome of *A. racemosus.* Previous studies reported that initial reactions of isoprenoid biosynthetic pathway occur in the leaves, while later step modifications and storage of saponins occurs in the roots [54] thus the amount of saponins is higher in roots. Mevalonate is the key precursor for synthesis of cholesterol and related isoprenoid compounds. Synthesis of mevalonate starts from acetyl CoA. The conversion of acetyl CoA to acetoacetyl CoA and then to 3-hydroxy-3-methylglutaryl CoA (3-HMG CoA) by HMG Co-A synthase (2 transcripts upregulated) corresponds to the biosynthetic pathway for ketone bodies. In the next step, the 3-HMG group is cleaved from the CoA and at the same time reduced to mevalonate with the help of (NADPH + H+), 3- HMG CoA reductase which is the key enzyme in cholesterol biosynthesis. *A. racemosus* transcriptome analysis has identified five different forms of 3-HMG CoA reductase and it was found to be specifically expressing in root tissue. Oxidosqualene which is formed by the action of the Squalene epoxidase enzyme from Squalene is the precursor in the biosynthesis of both triterpene and steroidal saponins in plant. We have identified two contigs showing homology with squalene epoxidase during transcriptome analysis, real time analysis further showed the specific expression of these two transcripts in root tissue only. From squalene upto steroidal sapogenin formation a total of 21 transcripts were identified from *A. racemosus* leaf and root transcriptome analysis (Additional file 3: Table S7).

### Methyl jasmonate treatment

Methyl Jasmonate plays critical roles in plant metabolism by up-regulating the expression of genes related to secondary metabolite biosynthesis. We studied transcriptional changes in leaf and root tissues of *A. racemosus* after methyl jasmonate (MeJA) treatment. In RT-PCR analysis transcripts of UGTs and CyP450 involved in glycosylation and oxygenation steps respectively, have shown induced accumulation after the treatment. The upregulation of these enzymes in response to MeJA treatment may be due to the fact that mono-oxygenases that catalyze oxygenation reactions and glycosyltransferases that catalyze the transfer of sugar molecules to steroidal compounds may produce diverse saponins in different conditions (stress) and control the activities of plants. Similar stimulatory effects of MeJA on the biosynthetic pathway of other triterpenoid saponin compounds have already been reported [55]. It is also reported that transcripts encoding the key triterpene biosynthetic enzyme β-amyrin synthase increased up to 50-fold by introducing MeJA to cell suspension cultures of *M. truncatula* [56]. It is known that Jasmonic acid upregulates the expression of defense-related genes, so it may be possible that saponin biosynthesis is also related to plant defense responses [57].

**Figure 6:**
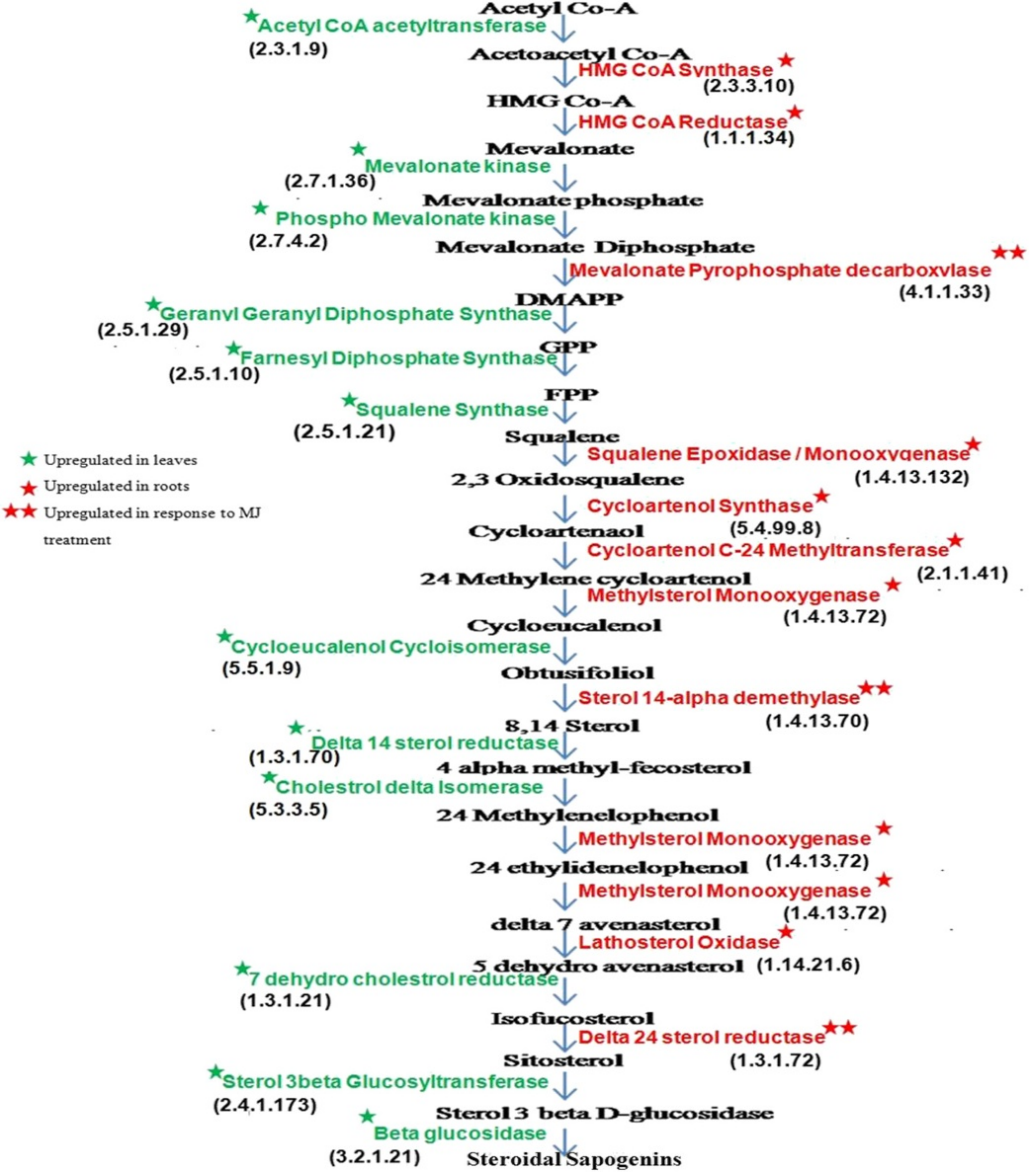
**Schematic representation of the sapogenin biosynthetic pathway.** Enzymes were highlighted and marked according to their specific expression in root tissue along with their up regulation after exogenous MeJA application in *A. racemosus*. The relative expression of representative gene transcripts was represented in comparison to actin taken as an internal control.

The increased expression of Mevalonate pyrophosphate decarboxylase, an important enzyme of Mevalonate pathway of saponin biosynthesis, in leaf after MeJA treatment further supported the fact that initial reactions of isoprenoid biosynthesis occur in the leaves. The increased expression of these transcripts in response to MeJA showed that saponin biosynthesis may be increased when the plant recognizes certain signaling molecules (elicitors) under stressed conditions. The identified transcripts involved in the steroidal biosynthetic pathway and their specific expression for root and leaf tissue in response to exogenous MeJA treatment is shown in Figure 6.

## Conclusion

The *de novo* transcriptome analysis of this very important Indian medicinal ayurvedic herb brings out for the first time novel transcripts related to saponin biosynthesis which has anticancer and anti-oxidant properties. The knowledge of intermediate transcripts identified in this study their functional characterization at biochemical, cellular and molecular level will be useful to metabolically engineer and understand the saponin biosynthetic pathway and its regulation in plants.

## Methods

### Plant material

Field grown plants of *A. racemosus* (CIM-SHAKTI), from the experimental plot of CSIR-CIMAP (Lucknow) field was used for transcriptome analysis. Leaf and root tissues from 2 month old healthy plants were harvested in the spring season and were stored at −80°C until used. These samples were further used for RNA extraction.

### RNA extraction and cDNA library construction

TRIzol method was used for RNA isolation from the root and leaf tissues. Transcriptome library for sequencing was constructed according to the Illumina TruSeq RNA library protocol outlined in “TruSeq RNA sample preparation Guide” (Part # 15008136; Rev.A; Nov 2010). Briefly 2.5 μg of total RNA by Qubit was enriched for Poly-A using RNA Purification Beads provided with the kit, enriched RNA was fragmented for 4 minutes at elevated temperature (94°C) in the presence of divalent cations and reverse transcribed with Superscript II Reverse transcriptase by priming with Random Hexamers. Second strand cDNA was synthesized in the presence of DNA polymerase I and RnaseH. The cDNA was cleaned up using Agencourt Ampure XP SPRI beads (Beckman Coulter). Illumina adapters were ligated to the cDNA molecules after end repair and addition of “A” base. SPRI cleanup was performed after ligation. The library was amplified using 11 cycles of PCR for enrichment of adapter ligated fragments. The prepared library was quantified using Nanodrop and validated for quality by running an aliquot on a High Sensitivity Bioanalyzer Chip (Agilent).

### *De novo*assembly and clustering

The leaf and root cDNA library was sequenced using paired end Illumina Hi-seq GAII Analyzer/454 GS FLX/5500 SOLiD System. QC and raw data processing were done by SeqQC-V2.1. Raw reads were cleaned by removing Vector (Adapter/Primer) contaminated reads. Empty reads and reads with unknown sequences ‘N’ was removed before data analysis. Contig assembly was carried out using a de Bruijn graph based *de novo* genome assembler Velvet_1.2.10 (https://www.ebi.ac.uk/~zerbino/velvet/) with a hash length 47 (Leaf Sample) and 43 (Root Sample). Velvet takes in short reads and assembles them into contigs using paired-end information. A draft assembly was built with hash length of 47 and 43 for leaf and root respectively. This draft assembly was used by observed-insert-length.pl and estimate-exp_cov.pl (from Velvet package) to estimate insert length and expected coverage parameters, which were then used to generate a final assembly. After this Oases_0.1.21 (https://www.ebi.ac.uk/~zerbino/oases/) use dynamic filters to improve the quality of the assembly with a hash length 47 (Leaf Sample) and 43 (Root Sample), and clusters them into small groups (loci). It then uses paired end information to construct transcript isoforms. The transcripts from three individual assemblies were clustered (CD-HIT v4.5.4 http://www.bioinformatics.org/cd-hit/) in order to generate a comprehensive reference. Sequence identity threshold and alignment coverage (for the shorter sequence) were both set at 80% to generate clusters.

### Sequence annotation and functional characterization

The contigs and singletons of leaf and root libraries were annotated using BLASTX program against NCBI database and all unigenes were utilized for homology searches against various protein databases such as NCBI nr (http://www.ncbi.nlm.nih.gov/), Swissprot (http://www.expasy.ch/sprot/), and the KEGG pathway (http://www.genome.jp/kegg/) with BLAST program (E-value < 1E-5), and the best aligning results were selected to annotate the unigenes. If the aligning results from different databases are in conflict with each other, the results from nr database were preferentially selected, followed by Swissprot, KEGG database. To further annotate the unigenes in this research, the Blast2GO program was used to get GO annotation according to molecular function, biological process and cellular component ontologies (http://www.geneontology.org/). Secondary metabolic Pathway assignments were performed according to the KEGG pathway database.

To assign functions to each unigene, gene ontology (GO) analysis was performed which classified unigenes of both root and leaf samples under the categories of Cellular component, Molecular Function and Biological Process. Each annotated sequence may have more than one GO term, either assigned in the different GO categories (Biological process, Molecular function and Cellular Component) or in the same category.

### Simple sequence repeats (SSRs) identification

All the contigs and singletons of leaf and root assemblies were used in a microsatellite program (MISA) (http://pgrc.ipk-gatersleben.de/misa/misa.html) for identification of SSR motifs. We searched for microsatellites from mononucleotide to hexa-nucleotide. The parameters used for simple sequence repeats were at least 6 repeats for di- and 5 for tri-, tetra, penta and hexa- nucleotide. Both perfect (i.e. contain a single repeat motif) and compound repeats (i.e. composed of two or more motifs separated by 100 bases) were identified.

### Digital gene expression profiling

To estimate the level of transcription for each gene, the number of reads that mapped within each annotated coding sequence (CDS) was determined. To enable comparison of expression levels, between two different RNA-seq experiments and different genes within the same experiment, it is necessary to normalize the read counts. The number of reads per kb of transcript per million mapped reads (RPKM) has been proposed as a useful metric that normalizes for variation in transcript length and sequence yield. Unigene expression levels were calculated as


Where C is the number of reads that uniquely aligned to one unigene; N is the total number of reads that uniquely aligned to all unigenes; L is the base number in the CDS of one unigene.

Digital gene expression was assessed using DESeq. DESeq allows a P-value to be determined in the absence of any available biological replicates, by treating the two conditions as replicates, under the assumption that only a small proportion of transcripts are differentially expressed. P-values were calculated under this assumption, and adjusted for multiple testing using the false discovery rate controlling procedure. In our analysis, DESeq results were filtered by P-value (<=0.05) and the absolute value of log2Ratio ≥ 1**.** P value formula was


where N is the number of all genes with GO annotation; n is the number of DGEs in N; M is the number of all genes that are annotated to the certain GO terms; m is the number of DGEs in M. Fold change values were calculated as Treated/Control expression values. Up and Down regulation was based on Log2FoldChange values (>1 Up, <−1 Down).

DGEs were also used in pathway enrichment analysis. We calculated the gene numbers in each pathway by mapping all DGEs to KEGG database (http://www.genome.jp/kegg).

### Transcription factor analysis

Transcription factors were predicted according to protein sequences obtained from CDS prediction. We used hmmsearch to search the domain of the plant transcription factors (http://plntfdb.bio.unipotsdam.de/v3.0/) and classified unigenes according to the gene family information.

### Quantitative Real-Time PCR (qRT-PCR) analysis

Twenty seven up regulated transcripts in root with potential roles in secondary metabolite biosynthesis were chosen for validation using qRT-PCR (primers designed for qRT-PCR analysis was shown in Additional file 5: Table S1). Field grown two months old *Asparagus racemosus* plants were harvested and washed with DEPC treated water. The leaf and root tissues were dried and separated before freezing in liquid nitrogen and were stored at −80°C until used. Total RNA was extracted from leaf and root tissues separately by RNeasy Mini Kit (Qiagen, USA).

In order to study the role of MeJA on the expression of potential transcripts related to steroidal saponin biosynthesis, 16 out of 27 transcripts were selected. Two months old field grown *A. racemosus* plants were used for MeJA treatment. A solution was prepared in DMSO and Triton-X containing 250 μM MeJA. The treatment was given by spraying the solution on aerial parts of the plant for 2–3 minutes; plants were sprayed again after 1 hour with the similar solution. For control, plants were sprayed with the solution containing only DMSO and Triton- X. After spraying the samples were covered with perforated autoclavable bags to maintain proper transpiration. After 3, 5 and 12 hours of treatment the samples were collected and washed properly with MQ to remove any contaminant or soil. Total RNA of leaf and root samples was extracted separately by RNeasy Mini Kit (Qiagen, USA) according to manufacturer's instructions. The RNA was quantified using Nanodrop and validated for quality by running an aliquot on a High Sensitivity Bioanalyzer Chip (Agilent). Approximately 1 μg of total RNA of each sample was converted into single-stranded cDNA using High Capacity cDNA Reverse Transcription Kit (Applied Biosystems). The cDNA products were then diluted 100-fold with deionized water. The reaction was performed on a 7500 FAST Real-Time PCR System (Applied Biosystems, USA) using the RealMasterMix (SYBR Green, Applied Biosystems). Expression levels of the selected unigenes were normalized to that of Actin, an internal reference gene. The relative expression is determined by raising 2 to the power of the negative value of ∆∆Ct for each sample. All the experiments were repeated using three biological and three experimental replicates and the data were analyzed statistically.

## Electronic supplementary material

Additional file 1: Figure S1: Length distribution of unigene sequences obtained after removal of adapter sequences from the de novo assembly. The unigenes are grouped from shortest to longest with each column representing the number of unigenes of that specific length. The maximum unigenes obtained after assembly were of an average size of more than 1200 read bp. **Figure S2.** Venn diagram for number of unigenes showing sequence homology with Arabidopsis and Liliopsida species (E < 0.00001). **Figure S3.** Frequency distribution of SSRs based on motif type obtained. (A) Leaf SSRs and (B) Root SSRs based on motif types. **Figure S4.** Distribution and expression of transcription factors among the uigenes obtained. (A) DGEs for every gene family transcription factor obtained in leaf and root tissue showing their up and down regulation. **Figure S5.** Fold expression of unigenes in root tissue in comparison with leaf tissue. The figure represents the fold change with number of unigenes correspond to this fold change. **Figure S6.** (a) Upregulation of specific family of transcription factors identified after leaf and root transcriptome based on DGE data (b) The distribution of transcription factors according to the gene family information and GO for both leaf and root tissues. (PPT 726 KB)

Additional file 2: Table S3: List of SSRs identified in leaf tissues with their type, pattern, size, starting and end points in transcript. (XLS 3 MB)

Additional file 3: Table S4: List of SSRs identified in root tissues with their type, pattern, size, starting and end points in transcript. **Table S5.** List of SSRs identified in saponin biosynthetic pathway genes. **Table S6.** List of the transcript IDs that are upregulated in root as compared to leaf. **Table S7.** List of enzymes related to Sapogenin biosynthetic pathway with their EC numbers that are upregulated in root of *A. racemosus*. **Table S8.** List of tissue specific unigenes related to Terpenoid biosynthetic pathway that is expressed in the root vs leaf transcriptome. (XLS 4 MB)

Additional file 4: Table S2: List of tissue specific unigenes related to KEGG pathway that is expressed in the root versus leaf transcriptome. (XLS 23 KB)

Additional file 5: Table S1: Primer sequences used in qRT-PCR analysis. (XLS 20 KB)
