# Supplementary material for: De novo leaf and root transcriptome analysis identified novel genes involved in Steroidal sapogenin biosynthesis in Asparagus racemosus
Source: BMC Genomics. 2014 Aug 30;15(1):746. doi: 10.1186/1471-2164-15-746 (PMC4162912; doi:10.1186/1471-2164-15-746)
Supplement: Supplementary file 1 — Additional file 1: Figure S1: Length distribution of unigene sequences obtained after removal of adapter sequences from the de novo assembly. The unigenes are grouped from shortest to longest with each column representing the number of unigenes of that specific length. The maximum unigenes obtained after assembly were of an average size of more than 1200 read bp. Figure S2. Venn diagram for number of unigenes showing sequence homology with Arabidopsis and Liliopsida species (E < 0.00001). Figure S3. Frequency distribution of SSRs based on motif type obtained. (A) Leaf SSRs and (B) Root SSRs based on motif types. Figure S4. Distribution and expression of transcription factors among the uigenes obtained. (A) DGEs for every gene family transcription factor obtained in leaf and root tissue showing their up and down regulation. Figure S5. Fold expression of unigenes in root tissue in comparison with leaf tissue. The figure represents the fold change with number of unigenes correspond to this fold change. Figure S6. (a) Upregulation of specific family of transcription factors identified after leaf and root transcriptome based on DGE data (b) The distribution of transcription factors according to the gene family information and GO for both leaf and root tissues. (PPT 726 KB) [file 12864_2014_6432_MOESM1_ESM.ppt]

## Slide 1
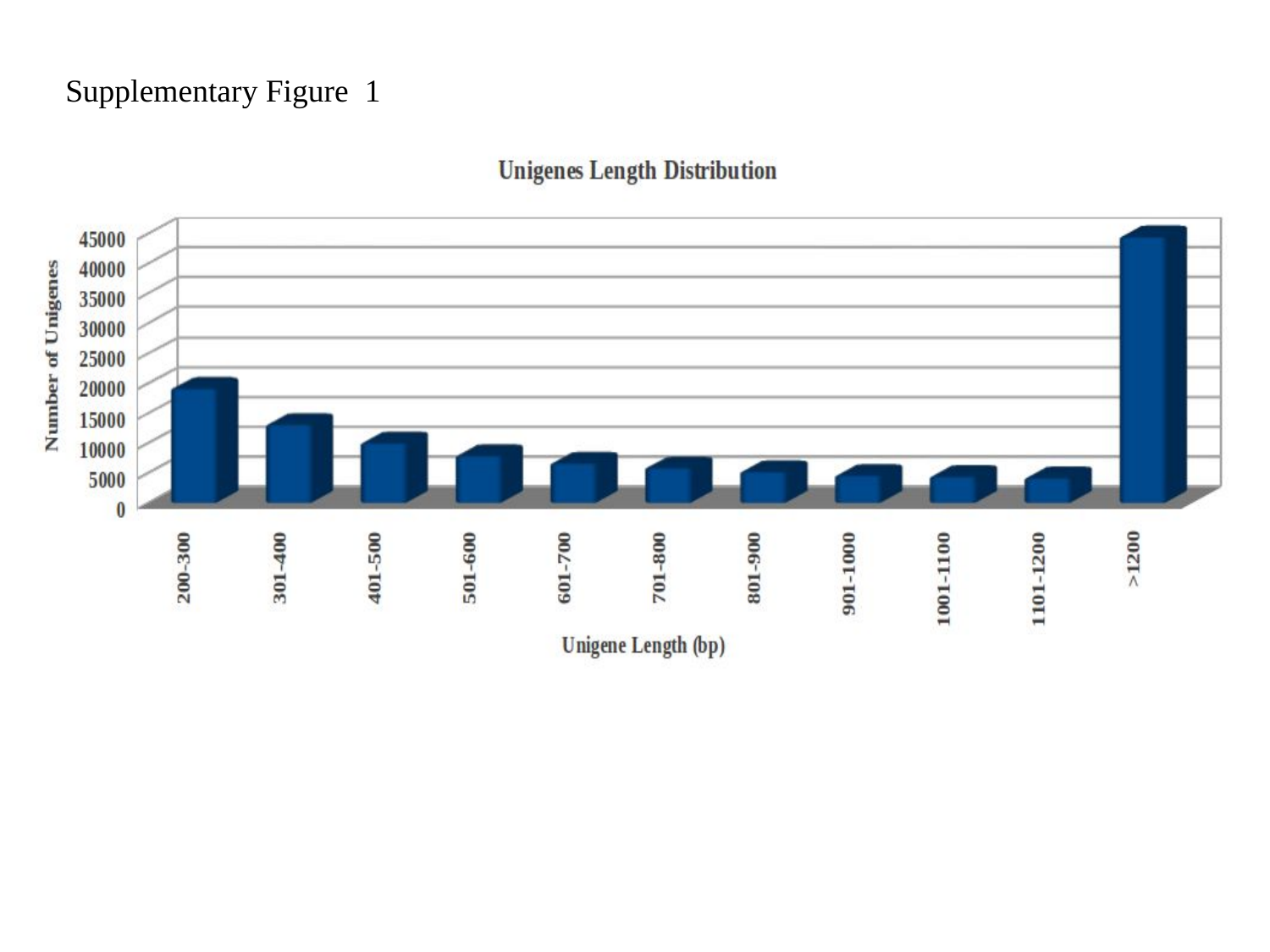

Supplementary Figure 1

## Slide 2
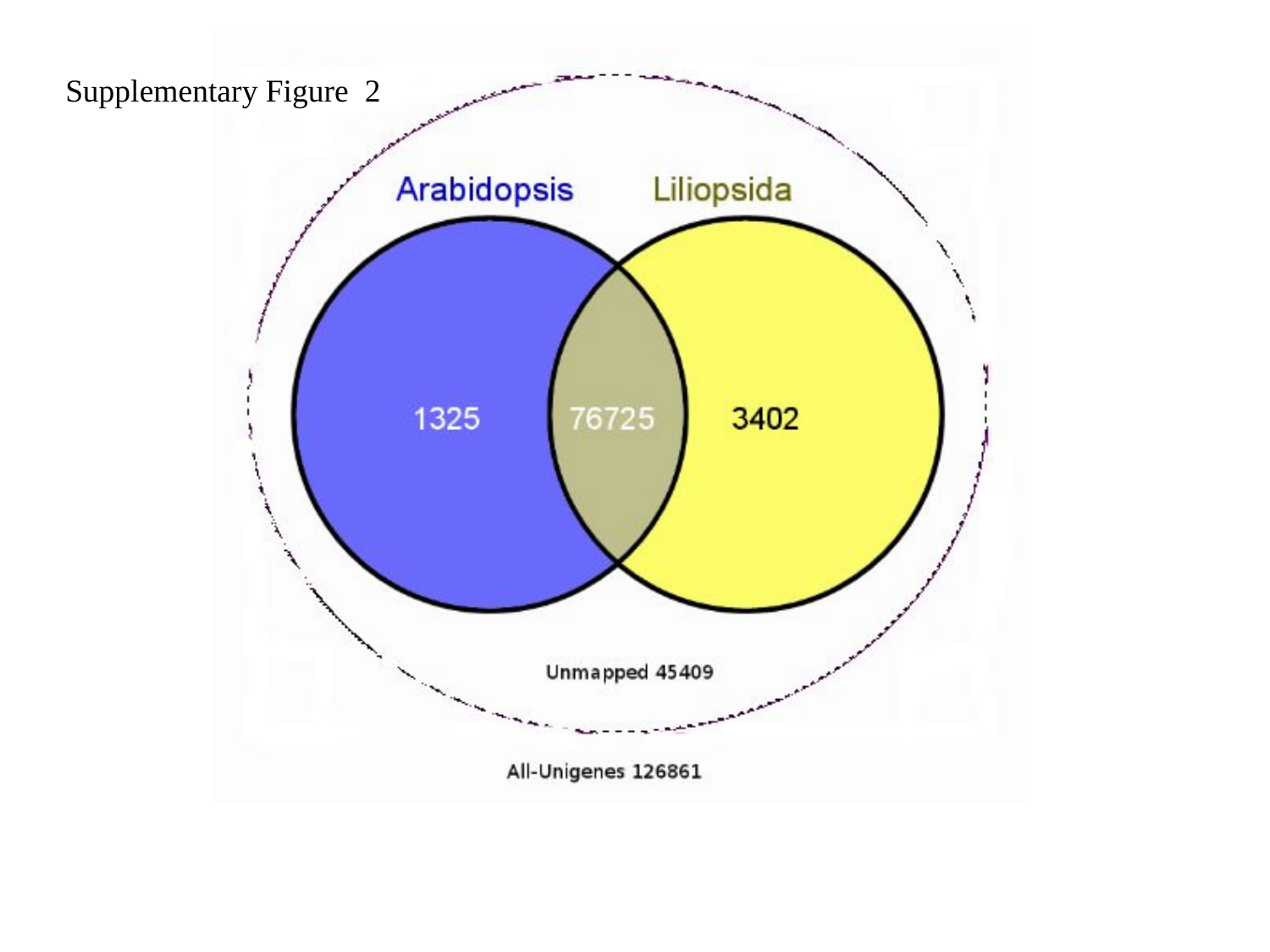

Supplementary Figure 2

## Slide 3
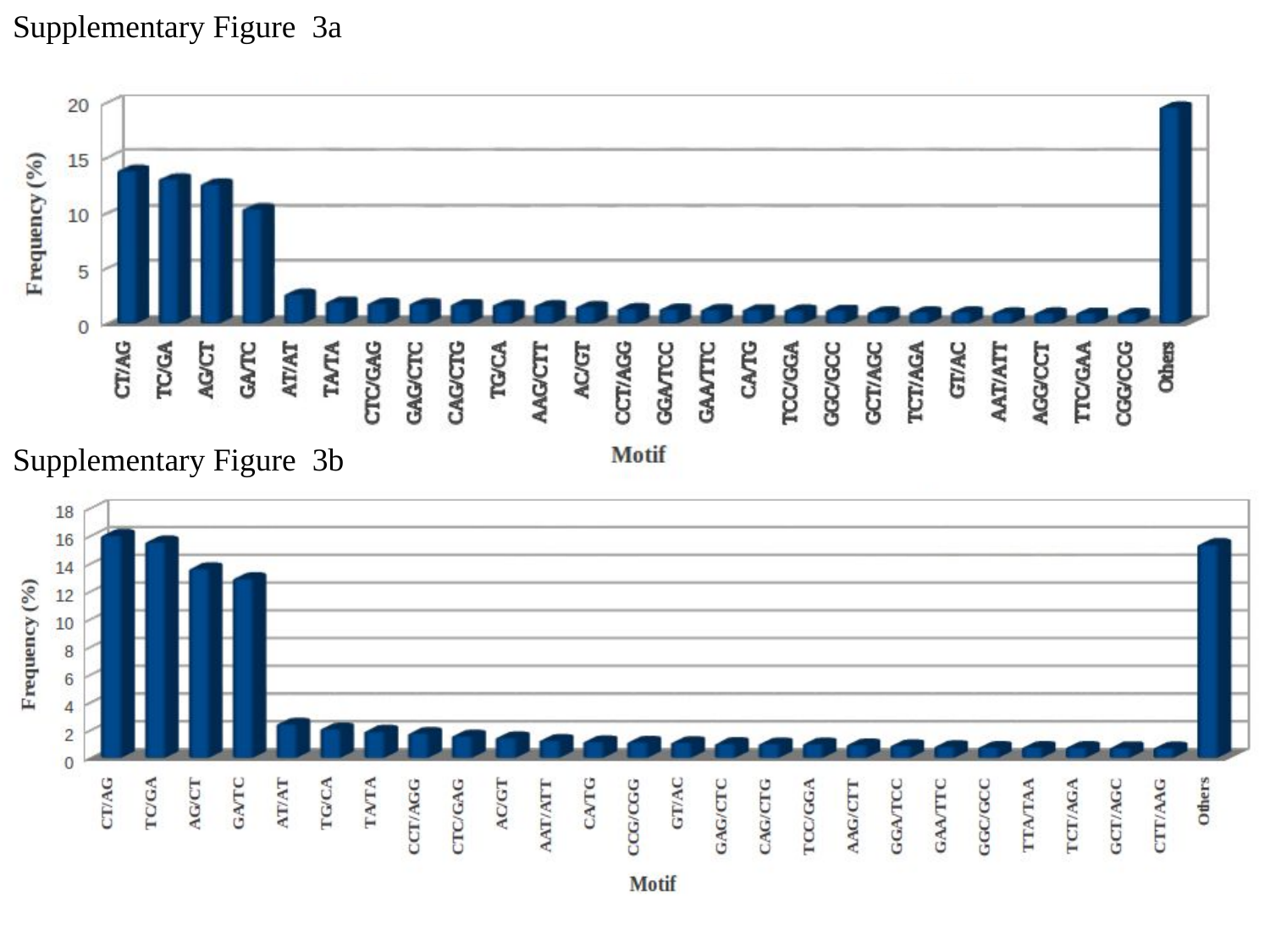

Supplementary Figure 3a
Supplementary Figure 3b

## Slide 4
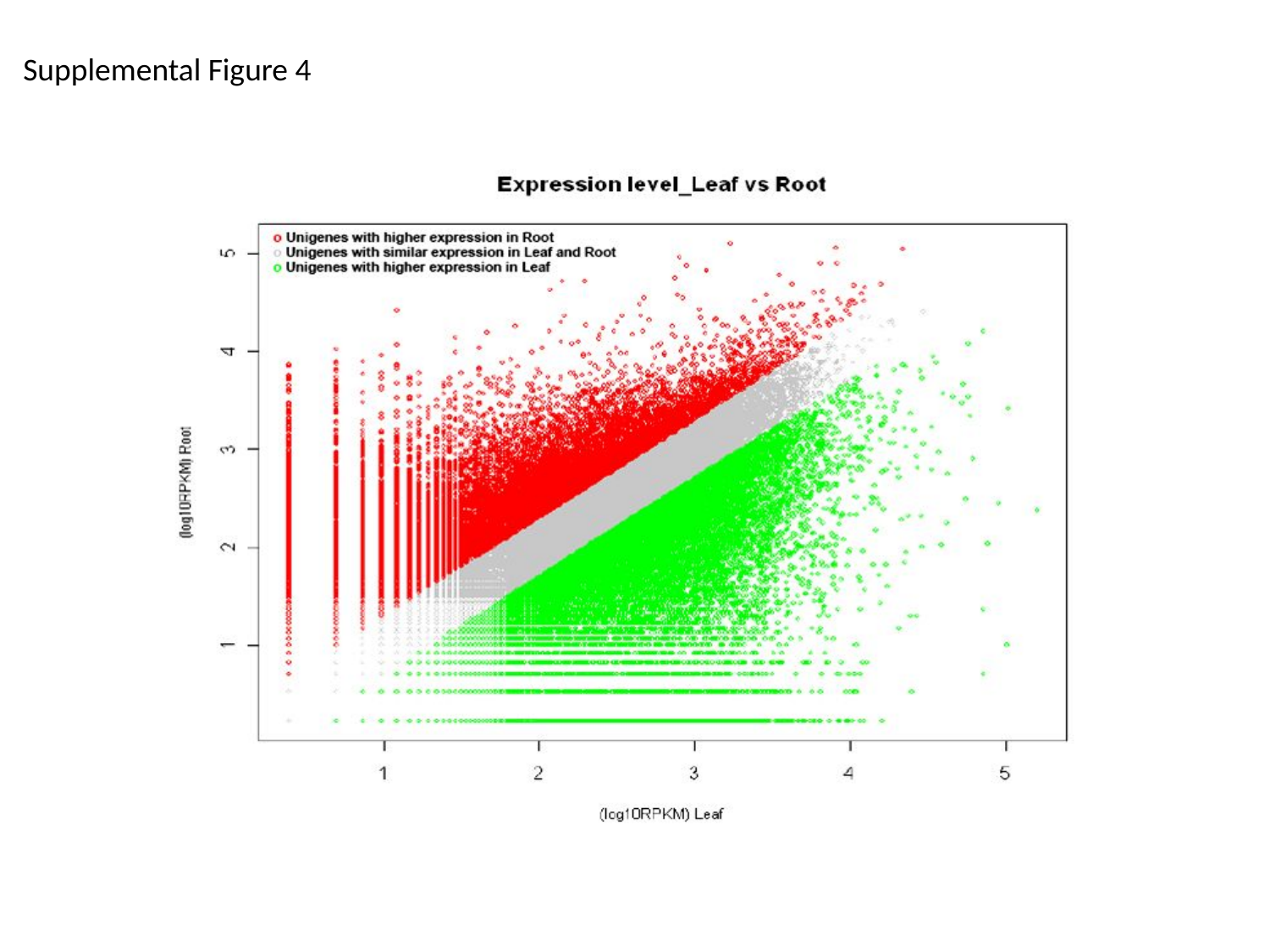

Supplemental Figure 4

## Slide 5
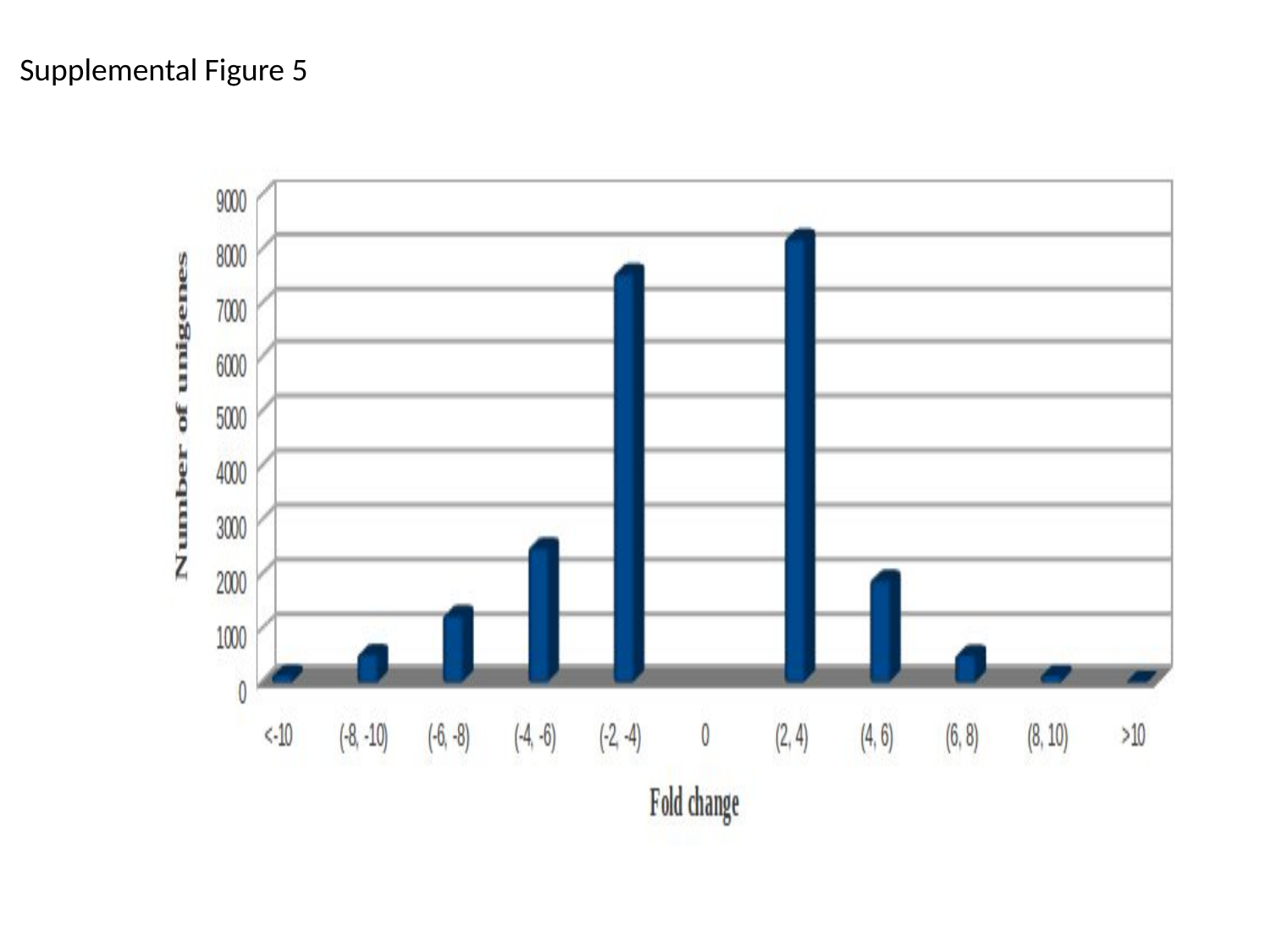

Supplemental Figure 5

## Slide 6
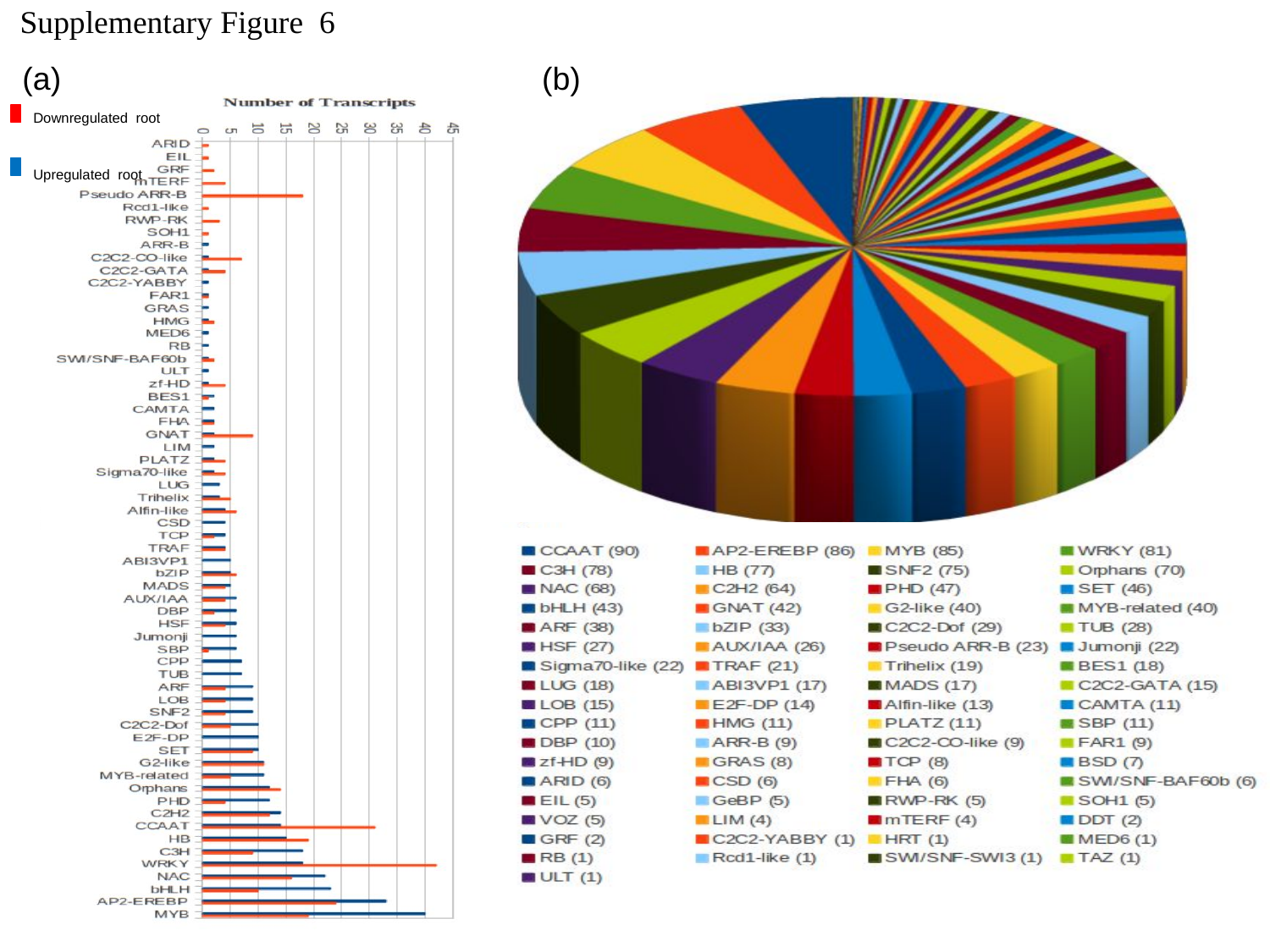

Supplementary Figure 6
(a)
(b)
Downregulated root
Upregulated root
